# Effect of immune checkpoint inhibitor therapy on biventricular mechanics in cancer patients over a short-term follow-up: a systematic review

**DOI:** 10.3389/fimmu.2025.1576175

**Published:** 2025-06-06

**Authors:** Andrea Sonaglioni, Antonino Bruno, Gian Luigi Nicolosi, Emanuela Fossile, Cristian Rubuano, Riccardo Ricotta, Maria Gemelli, Michele Lombardo, Paola Muti, Barbara Bassani

**Affiliations:** ^1^ Division of Cardiology, Istituto di ricovero e cura a carattere scientifico (IRCCS) MultiMedica, Milan, Italy; ^2^ Laboratory of Innate Immunity, Unit of Molecular Pathology, Biochemistry and Immunology, Istituto di ricovero e cura a carattere scientifico (IRCCS) MultiMedica, Milan, Italy; ^3^ Laboratory of Immunology and General Pathology, Department of Biotechnology and Life Sciences, University of Insubria, Varese, Italy; ^4^ Division of Cardiology, Policlinico San Giorgio, Pordenone, Italy; ^5^ Division of Oncology, Istituto di ricovero e cura a carattere scientifico (IRCCS) MultiMedica, Milan, Italy; ^6^ Medical Oncology Unit, Istituto di ricovero e cura a carattere scientifico (IRCCS) MultiMedica, Milan, Italy; ^7^ Istituto di ricovero e cura a carattere scientifico (IRCCS), MultiMedica, Milan, Italy; ^8^ Department of Biomedical, Surgical and Dental Sciences, University of Milan, Milan, Italy

**Keywords:** immune checkpoint inhibitor therapy, subclinical myocardial dysfunction, biventricular mechanics, cardiotoxicity, myocarditis

## Abstract

**Background:**

Immune checkpoint inhibitors (ICI) have demonstrated a survival benefit in various cancer types. A few numbers of imaging studies have recently measured myocardial strain parameters in cancer patients before and after ICI treatment, reporting not univocal results. This systematic review has been primarily designed to summarize the main findings of these studies and to examine the overall effect of ICI therapy on biventricular mechanics in cancer patients.

**Methods:**

All imaging studies evaluating the effect of ICI therapy on biventricular mechanics in cancer patients, selected from PubMed and EMBASE databases, were included. Imaging studies that analyzed myocardial strain parameters in highly selected cancer patients with ICI-related myocarditis were excluded. Relative change (RC) (%) from baseline of conventional and innovative indices of biventricular function was determined. Prevalence of cardiovascular complications was also assessed.

**Results:**

The full-texts of 12 studies with 554 ICI-treated cancer patients were analyzed. Myocardial strain parameters were measured by two-dimensional-speckle tracking echocardiography (STE) in seven studies, three-dimensional STE in two studies and cardiac magnetic resonance feature tracking in the remaining three studies. Average duration of follow-up was 3.1 months (range 0.5-7.3 months). All conventional indices of biventricular mechanics showed small and not statistically significant change after ICI treatment (RC ranging between -6.9 and +4.8%). Conversely, average left ventricular (LV)-global longitudinal strain (GLS), LV-global circumferential strain, LV-global radial strain, left atrial reservoir strain, right ventricular (RV)-GLS, RV-free wall longitudinal strain and right atrial reservoir strain were significantly worsened after ICI treatment (RC ranging between -9 and -19.2%). A definite cancer therapy-related cardiac dysfunction (CTRCD) was detected in 28.3% of ICI-treated patients (range 19.4-38.1%). The pooled prevalence of acute ICI-related myocarditis was 0.8% (range 0-4.6%) over follow-up period. Three out of seven ICI-related myocarditis patients (42.8%) were diagnosed with fulminant acute myocarditis.

**Conclusions:**

ICI treatment causes a significant deterioration of biventricular mechanics, early diagnosed by strain imaging methods. Myocardial strain parameters are more sensitive than conventional indices of systolic function for the early detection of subclinical ICI-related cardiotoxicity.

**Systematic review registration:**

https://www.crd.york.ac.uk/prospero/, identifier INPLASY202490131.

## Graphical Abstract

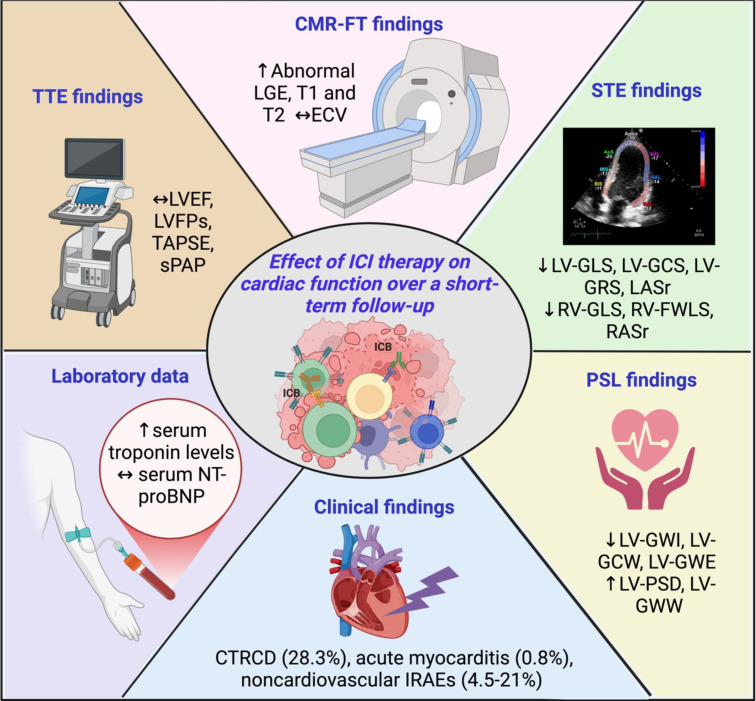

## Introduction

Immune checkpoint inhibitors (ICI), a major class of immuno-oncology therapeutics, have demonstrated a survival benefit in various cancer types, both in (neo)adjuvant and metastatic settings (1, 2). Differently from conventional anti-tumor therapies, ICI stimulate and enhance host immunity to eliminate cancer cells (3). As of April 2022, the U.S. Food and Drug Administration (FDA) has approved therapies targeting 3 immune checkpoints: cytotoxic T lymphocyte associated antigen 4 (CTLA-4), programmed death-1 (PD-1) and its ligand (PD-L1), and lymphocyte-activation gene 3 (LAG-3), for use as anticancer agents, either as monotherapy or in combination with other ICI or with chemotherapy and/or targeted therapy. ICI are approved for the treatment of melanoma, non-small cell lung cancer, classical Hodgkin lymphoma, head and neck squamous cell carcinoma, urothelial carcinoma, and renal cell carcinoma. Despite their advantages in cancer therapy, ICI may induce several immune-related adverse events (IRAEs), which may also affect the cardiovascular system (4–6). The ICI-related myocarditis accounts as the most severe type of IRAEs, that seems to most likely occur within the first 3 months, following treatment initiation, with a prevalence of 0.09–2.4% (7–9) and a mortality rate as high as 25–50% (10–12). Awareness of ICI-related myocarditis has led the researchers to evaluate less severe or even subclinical forms of ICI-related myocarditis (13). Left ventricular ejection fraction (LVEF), assessed by conventional transthoracic echocardiography (TTE), is not sufficiently sensitive for the detection of early changes in cardiac function (14, 15). Indeed, a decline in myocardial function might already occur even though LVEF is still normal. Speckle tracking echocardiography (STE) has been recently developed to overcome LVEF limitations in detecting subclinical myocardial dysfunction (16). This innovative imaging modality measures the deformation (strain) of myocardial fibers in systole and diastole, in longitudinal, circumferential and radial directions and the rate at which this deformation occurs (strain rate) (17). Myocardial deformation properties of both ventricles and atria may be accurately assessed by strain echocardiographic imaging. Early impairment in left ventricular (LV) global longitudinal strain (GLS), the most commonly used STE-derived index of myocardial contractility, has been found to occur before LVEF reduction in various clinical settings (18–20). Moreover, the myocardial strain of both ventricles and atria has been shown to correlate with the degree of myocardial edema and fibrosis, assessed by endomyocardial biopsy (21, 22). During the last few years, a few imaging studies have measured myocardial strain parameters in cancer patients undergoing ICI therapy before treatment and over a short-term follow-up period, reporting not univocal results. These studies evaluated myocardial deformation properties of ICI-treated patients by using TTE implemented with STE analysis or cardiac magnetic resonance feature tracking (CMR-FT). This systematic review has been primarily designed to summarize the main findings of these studies and to examine the overall effect of ICI therapy on biventricular mechanics in cancer patients. The pathophysiological mechanisms underpinning biventricular strain impairment in these patients will be discussed as well.

## Methods

This systematic review was performed according to the Preferred Reporting Items for Systematic Reviews and Meta-analyses (PRISMA) guidelines (23) and was registered in INPLASY database (registration number INPLASY202490131).

### Search strategy

A comprehensive search of all imaging studies evaluating the effect of ICI therapy on biventricular mechanics in cancer patients, was carried out by two independent reviewers (A.S. and M.L.) through September 2024, by using Medline and EMBASE databases. The search strategy included the following terms: “Immune checkpoint inhibitors” OR “ICI therapy” AND “cardiac function” AND “cancer therapy-related cardiac dysfunction” OR “CTRCD” AND “biventricular mechanics” AND “left ventricular global longitudinal strain” OR “LV-GLS” AND “right ventricular global longitudinal strain” OR “RV-GLS” AND “right ventricular free wall longitudinal strain” OR “RV-FWLS” AND “two-dimensional transthoracic echocardiography” OR “three-dimensional transthoracic echocardiography” AND “speckle tracking echocardiography” AND “cardiac magnetic resonance feature tracking” OR “CMR-FT”. ICI-related cardiotoxicity was assessed by two-dimensional (2D)-TTE or three-dimensional (3D)-TTE implemented with 2D-STE or 3D-STE analysis respectively, or by CMR-FT, and relevant biomarkers, such as serum levels of high-sensitivity cardiac troponin T (hs-cTnT) and N-terminal pro-brain natriuretic peptide (NT-proBNP). Search was limited to full-text articles published in English. There was no limitation of time period.

### Eligibility criteria

All imaging studies evaluating the effect of ICI therapy on biventricular mechanics in cancer patients were included. Conversely, imaging studies conducted on cancer patients who did not undergo ICI treatment, imaging studies conducted on cancer patients scheduled for ICI therapy without biventricular mechanics assessment, imaging studies that analyzed myocardial strain parameters only before ICI treatment without follow-up data, imaging studies that measured myocardial strain parameters in highly selected cancer patients with ICI-related myocarditis, non-clinical articles, animal studies, duplicate articles, case reports, conference presentations, reviews, correspondences, editorials, letters without data, and abstracts, were excluded.

### Study selection and data extraction

Two reviewers (A.S. and M.L.) screened the databases according to the inclusion criteria and performed data extraction independently. Information concerning: 1) demographics (age and sex); 2) anthropometrics [body surface area (BSA) and body mass index (BMI)]; 3) prevalence of the most common cardiovascular risk factors (hypertension, smoking, type 2 diabetes mellitus and dyslipidemia); 4) previous history of coronary artery disease (CAD), atrial fibrillation (AF) and chronic kidney disease (CKD); 5) cancer type; 6) ICI regimen (PD-1 inhibitors, PD-L1 inhibitors, CTLA-4 inhibitors or dual therapy); 7) conventional TTE parameters, including cavity chambers cavity size, left ventricular filling pressures (LVFPs) expressed by the E/e’ ratio, biventricular systolic function assessed by left ventricular ejection fraction (LVEF) and tricuspid annular plane systolic excursion (TAPSE) respectively, and systolic pulmonary artery pressure (sPAP) as noninvasive index of pulmonary hemodynamics, before and after ICI therapy; 8) myocardial strain parameters assessed by STE or CMR-FT, before and after ICI therapy; 9) CMR findings, including myocardial edema, inflammation or fibrosis detected after ICI therapy; 10) serum levels of hs-cTnT and NT-proBNP, before and after ICI therapy; 11) prevalence of cancer therapy-related cardiac dysfunction (CTRCD) (defined as new relative decline in GLS by >15% from the baseline value or new LVEF reduction by ≥10 percentage points to an LVEF 40%–49%) (24), IRAEs and/or ICI-induced myocarditis over follow-up period; 12) finally, the current medical treatment; were independently collected by the two reviewers. Relative change (%) from baseline of all main TTE- and strain imaging-derived indices of biventricular mechanics was determined. A third author (G.L.N.) checked the extracted data for accuracy and resolved possible discrepancies between reviewers.

### Risk of bias assessment

**Figure 1:**
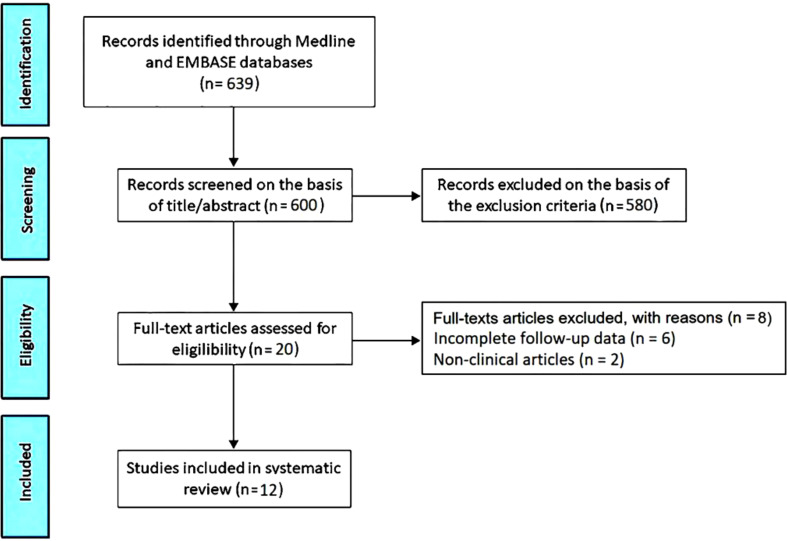
Flow diagram used for identifying the included studies.

Articles included in this systematic review were assessed for risk of bias (RoB) using the National Institutes of Health (NIH) Quality Assessment Tool for Observational Cohort and Cross-Sectional Studies (25). All the studies were assigned a “yes”, “no”, or “other” to each of the 14 criteria outlined in the appraisal tool. Then, by considering each criterion, the investigators evaluated the overall quality of the study and assigned an overall “good” (met 11–14 criteria), “fair” (met 6–10 criteria), “poor” (met 0–5 criteria) rating to each study. The quality rating was independently estimated by two authors (A.S. and G.L.N.). Disagreement was resolved by consensus. The PRISMA flow diagram used for identifying the included studies is depicted in **Figure 1**.

## Results

The initial search yielded a total of 639 studies. Of those, 39 (6.1%) were removed as duplicates. After screening titles and abstracts, a further 580 studies (90.8%) were removed, based on exclusion criteria. The evaluation of the full text of the remaining 20 studies (3.1%) resulted in further 8 exclusions (1.2%). A total of 12 studies (1.9%) (26–37) were thus included in this systematic review, totaling 554 ICI-treated cancer patients. Clinical characteristics and main findings of the included studies are summarized in **Table 1**.

**Table 1:** Summary and main findings of the included studies.

Study name, publication year and country	Population size	Sex (%)	Mean age (yrs)	Study design	Imaging method	F.U. (months)	ICI-induced effects on biventricular mechanics over F.U.		
							Increasing pattern	Decreasing pattern	Non-significant effect
Mylvaganam R et al. (2021), USA (26)	24	Males: 54 Females: 46	74 (52-85)	Retrospective, monocentric	2D-echo, CT	2.8	PA/Ao ratio	RV-FWLS	LVEF, TAPSE
Higgins AY et al. (2021), USA (27)	20	Males: 75 Females: 25	61 (51.4-70.6)	Retrospective, monocentric	CMR-FT	3	Abnormal LGE, T2	LV-GLS, LV-GRS	LVEF
Faron A et al. (2021), Germany (28)	22	Males: 59 Females: 41	65 (51-79)	Prospective, monocentric	CMR-FT	3	T1, T2	LV-GLS	LGE, ECV
Lee SH et al. (2022), Korea (29)	22	Males: 48.3 Females: 51.7	51 (37-65)	Prospective, monocentric	2D-echo	6	Serum troponin	s’, e’, LVEF; LV-GLS	
Pohl J et al. (2022), Germany (30)	30	Males: 60 Females: 40	59 (46-72)	Retrospective, monocentric	2D-echo	1		RV-FWLS, RASct	Serum NT-proBNP; LVEF, LV-GLS, FAC, sPAP
Liu J et al. (2022), China (31)	36	Males: 77.8 Females: 22.2	60.7 (51.5-69.9)	Prospective, monocentric	CMR-FT	3	RVIP-LGE	LV-GRS, RV-GRS	T1, T2, ECV LVEF, RVEF, LV-GLS
Quinaglia T et al. (2022), USA (32)	62	Males: 68 Females: 32	66 (51-81)	Retrospective, multicentric	2D-echo	1		LVEF; LV-GLS, LV-GCS, LV-GRS	
Tamura Y et al. (2022), Japan (33)	129	Males: 76 Females: 24	65.6 (54.4-76.8)	Retrospective, monocentric	2D-echo	0.46		LV-GLS; basal LS, mid LS	LVEF; apical LS
Xu A et al. (2022), China (34)	55	Males: 72.3 Females: 27.7	62 (50-74)	Prospective, monocentric	3D-ech	7.3		LVEF, TAPSE; LV-GLS, LV-GCS, RV-GLS, RV-FWLS	Serum troponin
Li X et al. (2023), China (35)	52	Males: 71 Females: 29	62.7 (53.5-71.9)	Prospective, monocentric	2D-echo	2.8	LV-PSD, LV-GWW	LV-GLS, LV-GWI, LV-GCW, LV-GWE	
Chunlan S et al. (2024), China (36)	43	Males: 62.8 Females: 37.2	56 (46-66)	Prospective, monocentric	2D-echo	4.2	LV-GWW	LV-GLS, LV-GWI, LV-GCW, LV-GWE	
Delombaerde D et al. (2024), Belgium (37)	59	Males: 76 Females: 24	68 (56-80)	Prospective, multicentric	3D-echo	3	Serum troponin		Serum NT-proBNP; LVEF, LV-GLS

2D, two-dimensional; 3D-three-dimensional; Ao, aorta; CMR-FT, cardiac magnetic resonance feature tracking; CT, computed tomography; ECV, extracellular volume; FAC, fractional area change; F.U., follow-up; FWLS, free wall longitudinal strain; GE, General Electric; GCS, global circumferential strain; GCW, global constructive work; GLS, global longitudinal strain; GRS, global radial strain; GWE, global work efficiency; GWI, global work index; GWW, global wasted work; LGE, late gadolinium enhancement; LS, longitudinal strain; LV, left ventricular; LVEF, left ventricular ejection fraction; NT-proBNP, N-terminal pro-B-type natriuretic peptide; PA, pulmonary artery; PSD, peak strain dispersion; RASct, right atrial contractile strain; RV, right ventricular; RVEF, right ventricular ejection fraction; RVIP, right ventricular insertion point; sPAP, systolic pulmonary artery pressure; STE, speckle tracking echocardiography; TAPSE, tricuspid annular plane systolic excursion.

The included studies were published between 2021 and 2024. Four studies were performed in China, three in the USA, two in Germany, one in Korea, Japan and Belgium. Mean age of cancer patients was 62.5 yrs (range 51–74 yrs). The average percentage of males was 66.7% (range 48.3-77.8%). Seven studies (58.3% of total) had a prospective design, whereas the remaining five (41.7% of total) were retrospective. The great majority of studies (83.3% of total) were monocentric, while only two studies (16.7% of total) involved more than one institution.

Myocardial strain parameters were assessed by 2D-STE in seven studies (58.3% of total), 3D-STE in two studies (16.7% of total) and CMR-FT in three studies (25% of total). Two Chinese studies (35, 36) investigated the effect of ICIs on LV myocardial work indices assessed by pressure-strain loop (PSL) analysis. Mylvaganam R et al. (25) performed Computed Tomography (CT) scan for evaluating the impact of ICIs on the development of subclinical pulmonary vascular disease in cancer patients. Half studies analyzed myocardial deformation indices by using a Philips software, four studies by a GE software and two studies by a Siemens software.

All cancer patients were examined before and after ICI administration. Average duration of follow-up period was 3.1 months (range 0.5-7.3 months). Baseline clinical characteristics of cancer patients are reported in **Table 2**. Cancer patients showed a moderate prevalence of smoking and hypertension and a low-to-moderate prevalence of dyslipidemia, type 2 diabetes, CAD, AF and CKD. The most common indications for ICI treatment were lung cancer (44.9%), melanoma (34.1%), bladder cancer (31.3%) and head and neck cancer (24.3%). Approximately two-third of patients were treated with PD-1 inhibitors, particularly pembrolizumab and nivolumab, whereas PD-L1 inhibitors and CTLA-4 inhibitors were administered in 19.1% and 4.8% of cancer patients. 20.2% of patients received combination ICI therapy. At basal evaluation, less than one-third of participants made regular use of antiplatelets and cardioprotective drugs, such as beta blockers, angiotensin converting enzyme inhibitors (ACEi) or angiotensin receptor blockers (ARBs) and statins.

**Table 2:** Baseline clinical characteristics of cancer patients before ICI treatment.

	Average value (IQR)	Number of studies for parameters assessed (%)
Demographics		
Age (yrs)	62.5 (51-74)	12 (100)
Male sex (%)	66.7 (48.3-77.8)	12 (100)
Anthropometrics		
BSA (m^2^)	1.7 (1.67-1.9)	4 (33.3)
BMI (Kg/m^2^)	24.9 (20.3-27.2)	7 (58.3)
Cardiovascular risk factors and cardiovascular disease burden		
Hypertension (%)	34.9 (15-63)	11 (91.7)
Current or prior smoking (%)	44.8 (14-63)	9 (75)
Type 2 diabetes (%)	12.4 (0-30)	10 (83.3)
Dyslipidemia (%)	21.1 (9-66)	7 (58.3)
CAD (%)	10.7 (3.4-19)	8 (66.7)
AF (%)	13 (3.4-27.4)	4 (33.3)
CKD (%)	15.8 (0-36)	5 (41.7)
Cancer type		
Lung cancer (%)	44.9 (4.5-100)	9 (75)
Melanoma (%)	34.1 (2.3-100)	8 (66.7)
Bladder (%)	31.3 (7-60)	3 (25)
Head and neck (%)	24.3 (3.2-65.9)	4 (33.3)
Renal cell carcinoma (%)	11.5 (4-25)	7 (58.3)
GI cancer (%)	10.1 (6.2-13.9)	2 (16.7)
Other (breast, liver, gynecologic cancer, lymphoma, sarcoma)	18.7 (1.8-100)	1 study for each cancer type
ICI regimen		
**PD-1 inhibitors (%)**	65.2 (0-89.1)	12 (100)
Pembrolizumab (%)	34.7 (0-54)	12 (100)
Nivolumab (%)	29.5 (0-53)	12 (100)
Cemiplimab (%)	0.9 (0-9.1)	12 (100)
**PD-L1 inhibitors (%)**	19.1 (0-100)	12 (100)
Atezolizumab (%)	6.1 (0-17)	12 (100)
Avelumab (%)	1.7 (0-19)	12 (100)
Durvalumab (%)	11.3 (0-100)	12 (100)
**CTLA-4 inhibitors (%)**	4.8 (0.47)	12 (100)
Ipilimumab (%)	3.7 (0-35)	12 (100)
Tremelimumab (%)	1.1 (0-12)	12 (100)
**Dual therapy (%)**	20.2 (0-50)	12 (100)
Current medical treatment		
Antiplatelets (%)	27 (22-30)	3 (25)
BB (%)	19 (4-37)	8 (66.7)
ACEi/ARBs (%)	21.6 (9-41)	7 (58.3)
CCB (%)	15.8 (8-25)	4 (33.3)
Diuretics (%)	13.6 (3.1-24)	2 (16.7)
Statins (%)	30.9 (12-49)	5 (41.7)

Data are expressed as median and IQR. ACEi, angiotensin converting enzyme inhibitors; AF, atrial fibrillation; ARBs, angiotensin receptor blockers; BB, beta blockers; BMI, body mass index; BSA, body surface area; CAD, coronary artery disease; CCB, calcium channel blockers; CKD, chronic kidney disease; CTLA-4, cytotoxic T lymphocyte associated antigen 4; GI, gastro-intestinal; ICI, Immune checkpoint inhibitors; IQR, interquartile range; PD-1, programmed death-1; PD-L1, programmed death-ligand 1.The bold texts represent the ICI macro-category.

Main echocardiographic data obtained in cancer patients before and after ICI treatment are described in **Table 3**. Among TTE parameters, the most commonly assessed were LVEF (measured by all the included studies) and E/e’ ratio (measured by half of the studies), while information concerning biventricular cavity sizes, RV systolic function and pulmonary hemodynamics were provided by a limited percentage of studies ranging from 16.7% and 41.7% of total. TTE performed before ICI treatment revealed normal cardiac chambers internal dimensions, normal biventricular systolic function, first degree of diastolic dysfunction, normal LVFPs and normal sPAP. All conventional echocardiographic parameters showed small and not statistically significant change after ICI treatment (relative change ranging between -6.9 and +4.8%). Indeed, all principal indices of biventricular systolic function (LVEF and TAPSE) and hemodynamics (LVFPs and sPAP) were within the normal range either before or after ICI treatment.

**Table 3:** Main conventional indices of cardiac morphology and function and myocardial strain parameters obtained in cancer patients before and after ICI treatment.

	Baseline	Follow-up	Relative change (%)	Number of studies for parameters assessed (%)
Conventional indices of cardiac morphology and function				
LVEDD (mm)	46.8 (46-47.5)	46.3 (44-48.6)	-1.1	5 (41.7)
LVEDV (ml)	110.8 (88-139)	108.9 (83-134.9)	-1.7	5 (41.7)
LAVi (ml/m^2^)	27.5 (25-30.4)	28.8 (24-34.8)	+4.7	4 (33.3)
LVEF (%)	61.1 (51.5-65)	59.3 (49.2-64.1)	-2.9	12 (100)
E/A ratio	0.96 (0.68-1.26)	0.97 (0.58-1.31)	+1	4 (33.3)
E/e’ ratio	8.4 (7-10.1)	8.8 (6.9-11)	+4.8	6 (50)
RV basal diameter (mm)	36.5 (35-38)	35 (33-37)	-4.1	2 (16.7)
FAC (%)	45.1 (38-52.2)	43.3 (38-48.6)	-4.0	2 (16.7)
TAPSE (mm)	21.7 (18.3-26)	20.2 (14.6-25)	-6.9	4 (33.3)
sPAP (mmHg)	27.3 (22-33)	27.7 (25-31)	+1.5	3 (25)
Myocardial strain parameters				
LV-GLS (%)	19.4 (13.4-23.4)	16.8 (11.1-21.3)	-13.4	11 (91.7)
LV-GCS (%)	23.4 (18-28.8)	20.2 (14.4-25)	-13.7	4 (33.3)
LV-GRS (%)	38.1 (31.9-44.5)	30.8 (23-37.8)	-19.2	3 (25)
LASr (%)	27.9 (27-28.8)	25.4 (24.5-26.3)	-9.0	2 (16.7)
RV-GLS (%)	16.6 (14.8-18.4)	14.2 (13.4-15)	-14.5	2 (16.7)
RV-FWLS (%)	23.9 (20.6-25.5)	20.1 (16.7-22.4)	-15.9	3 (25)
RASr (%)	41.4 (31.7-51)	36 (27.3-44.7)	-13.0	2 (16.7)

Relative change (%) describes the size of the absolute change in comparison to the baseline value. FAC, fractional area change; FWLS, free wall longitudinal strain; GCS, global circumferential strain; GLS, global longitudinal strain; GRS, global radial strain; ICI, Immune checkpoint inhibitors; LASr, left atrial reservoir strain; LAVi, left atrial volume indexed; LV, left ventricular; LVEDD, left ventricular end-diastolic diameter; LVEDV, left ventricular end-diastolic volume; LVEF, left ventricular ejection fraction; RASr, right atrial reservoir strain; RV, right ventricular; sPAP, systolic pulmonary artery pressure; TAPSE, tricuspid annular plane systolic excursion.

**Figure 2:**
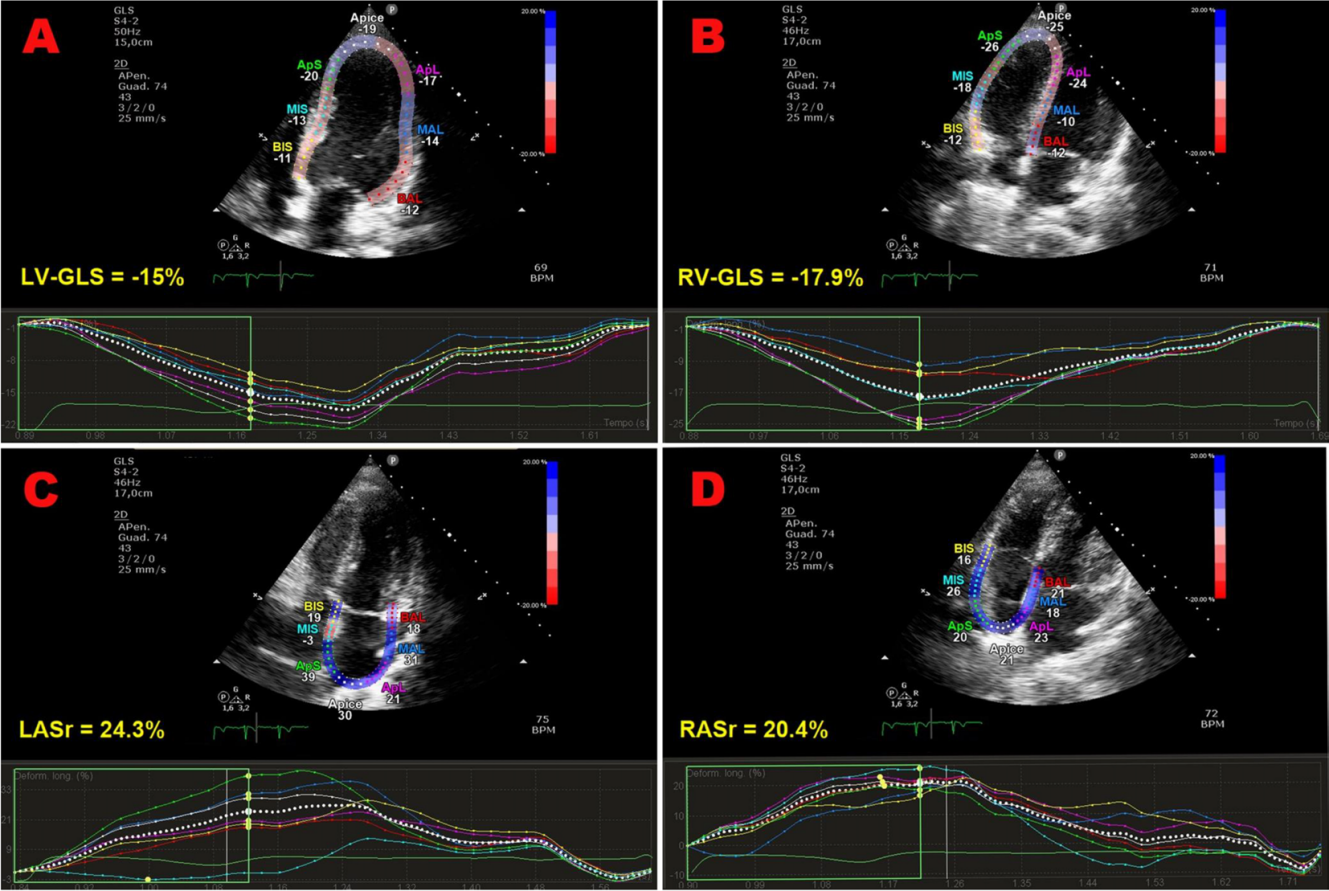
Representative examples of impaired biventricular and biatrial myocardial strain parameters obtained by strain echocardiographic imaging from the apical four-chamber view, in a ICI-treated cancer patient, over a short-term follow-up period. Absolute values of LV-GLS **(A)**, RV-GLS **(B)**, LASr **(C)** and RASr **(D)** were all moderately reduced in comparison to the accepted reference values. GLS, global longitudinal strain; ICI, Immune checkpoint inhibitors; LASr, left atrial reservoir strain; LV, left ventricular; RASr, right atrial reservoir strain; RV, right ventricular; STE, speckle tracking echocardiograp.

Strain imaging was primarily focused on LV-GLS assessment (measured by 91.7% of studies), while LV global circumferential strain (GCS) and LV global radial strain (GRS) were measured by 33.3% and 25% of studies, respectively. In addition, left atrial reservoir strain (LASr), RV-GLS, RV-FWLS and right atrial reservoir strain (RASr) were determined by less than one-third of studies, particularly those studies that analyzed myocardial strain parameters by using CMR-FT. Compared with the baseline data, average LV-GLS, LV-GCS, LV-GRS, LASr, RV-GLS, RV-FWLS and RASr were significantly worsened after ICI treatment (relative change ranging between -9 and -19.2%). The average magnitude of all strain parameters obtained during follow-up period was significantly lower in comparison to the accepted reference values (38–42). However, three Authors (30, 31, 37) did not observe any significant change in LV-GLS in ICI-treated patients. When comparing LV-GLS changes recorded at follow-up time point vs baseline, assessed by different imaging methods, CMR-FT studies detected a more pronounced LV-GLS deterioration in comparison to strain echocardiographic studies (relative change 21.2% vs 8.3%). Representative examples of impaired biventricular and biatrial myocardial strain parameters obtained by strain echocardiographic imaging from the apical four-chamber view in a ICI-treated cancer patient, over short-term follow-up period, are illustrated in **Figure 2**.

Differently from other Authors, Tamura Y et al. (33) performed an accurate analysis of global and regional longitudinal strain (LS) before and after ICI administration; they demonstrated an early relative worsening of ≥10% in the basal and mid LS and ≥15% in GLS, associated with increased hsTnI, in patients receiving ICIs.

PSL analysis highlighted that myocardial work indices, specifically, LV-GLS, LV-global work index (GWI), LV-global constructive work (GCW), and LV-global work efficiency (GWE) all decreased to varying degrees after ICI therapy, while LV- peak strain dispersion (PSD) and LV-global wasted work (GWW) increased (35, 36).

CMR-FT studies reported nonischemic late gadolinium enhancement (LGE) lesions (27) and increased T1 and T2 relaxation times (27, 28) after ICI treatment, while extracellular volume fraction (ECV) values of global myocardium at follow-up showed no significant differences compared with baseline values (28, 31). In addition, small pericardial effusions were detected in a limited percentage of participants (28).

Finally, the CT scan study performed by Mylvaganam R et al. (26) revealed a significant increase in the pulmonary artery to aorta (PA/Ao) ratio in patients who received anti-PD-1 therapy.

Four Authors (26, 31, 35, 36) measured the intraclass correlation coefficient (ICC) for evaluating the intra- and inter-rater reproducibility of myocardial strain parameters, myocardial work indices, CMR quantitative parameters and finally CT scan measurements, demonstrating excellent agreement, with ICC values >0.90.

Concerning laboratory data, serial assessment of serum Hs-TnT and NT-proBNP levels was performed by 66.7% and 25% of studies, respectively. ICI-treated patients were diagnosed with a moderate increase in serum Hs-TnT levels over follow-up period, whereas no significant changes in serum NT-proBNP levels were detected.

All noncardiovascular and cardiovascular IRAEs detected in ICI-treated cancer patients by the included studies are listed in **Table 4**. The prevalence of noncardiovascular IRAEs was accurately analyzed by five studies (26–28, 32, 35). The most common ones were pneumonitis, dermatitis, thyroiditis and myositis (with a pooled prevalence ranging from 7.5% and 21%), whereas colitis, hypophysitis, hepatitis and arthritis affected a lower percentage of patients ranging from 4 to 5%. With regards to the cardiovascular IRAEs, five studies (41.7% of total) did not report any cardiovascular toxicity at the follow-up time point. A definite CTRCD was detected in 28.3% of ICI-treated patients (range 19.4-38.1%). The pooled prevalence of acute ICI-related myocarditis was 0.8% (range 0-4.6%) over follow-up period. Three out of seven patients (42.8%) were diagnosed with fulminant acute myocarditis.

**Table 4:** Pooled prevalence of noncardiovascular and cardiovascular IRAEs in ICI-treated patients over follow-up period, detected by the included studies.

	Baseline	Follow-up	Number of studies for parameters assessed (%)
Noncardiovascular IRAEs			
Pneumonitis (%)	/	21 (15-27)	3 (25)
Dermatitis (%)	/	20.7 (17-27)	3 (25)
Thyroiditis (%)	/	10.7 (8-15)	3 (25)
Myositis (%)	/	7.5 (5-10)	2 (16.7)
Other (colitis, hypophysitis, hepatitis and arthritis) (%)	/	4.5 (4-5)	4 (33.3)
Cardiovascular IRAEs			
Serum Hs-TnT (ng/L)	8.9 (1.2-20)	31.1 (10-53.3)	8 (66.7)
Serum NT-proBNP (ng/L)	92.4 (20.1-131)	86.2 (24.7-119)	3 (25)
CTRCD (%)	/	28.3 (19.4-38.1)	12 (100)
Myocarditis (%)	/	0.8 (0-4.6)	12 (100)

CTRCD, cancer therapy-related cardiac dysfunction; IRAEs, immune-related adverse events; Hs-cTNT, high-sensitivity cardiac troponin T; ICI, Immune checkpoint inhibitors; NT-proBNP, N-terminal pro-brain natriuretic peptide.

### Risk of bias assessment

The NIH quality rating was estimated as fair for one study and good for eleven studies (**Table 5**). The Cohen’s Kappa coefficient for the agreement between the reviewers in the RoB assessment was 0.85, indicating a high level of agreement.

**Table 5:** Quality assessment of included studies.

NIH Quality Assessment Tool for Observational Cohort and Cross-Sectional Studies															
Study name	Q1	Q2	Q3	Q4	Q5	Q6	Q7	Q8	Q9	Q10	Q11	Q12	Q13	Q14	Quality
Mylvaganam R et al. (2021) (26)	Yes	Yes	Yes	Yes	No	Yes	Yes	Yes	Yes	Yes	Yes	Yes	Yes	Yes	13 (Good)
Higgins AY et al. (2021) (27)	Yes	Yes	Yes	Yes	No	Yes	Yes	No	Yes	Yes	Yes	NS	Yes	No	10 (Fair)
Faron A et al. (2021) 28)	Yes	Yes	Yes	Yes	No	Yes	Yes	No	Yes	Yes	Yes	Yes	Yes	No	11 (Good)
Lee SH et al. (2022) (29)	Yes	Yes	Yes	Yes	No	Yes	Yes	Yes	Yes	Yes	Yes	Yes	Yes	Yes	13 (Good)
Pohl J et al. (2022) (30)	Yes	Yes	Yes	Yes	No	Yes	Yes	Yes	Yes	Yes	Yes	NS	Yes	No	11 (Good)
Liu J et al. (2022) (31)	Yes	Yes	Yes	Yes	No	Yes	Yes	Yes	Yes	Yes	Yes	Yes	Yes	No	12 (Good)
Quinaglia T et al. (2022) (32)	Yes	Yes	Yes	Yes	No	Yes	Yes	No	Yes	Yes	Yes	Yes	Yes	Yes	12 (Good)
Tamura Y et al. (2022) (33)	Yes	Yes	Yes	Yes	No	Yes	Yes	No	Yes	Yes	Yes	Yes	Yes	Yes	12 (Good)
Xu A et al. (2022) (34)	Yes	Yes	Yes	Yes	No	Yes	Yes	Yes	Yes	Yes	Yes	NS	Yes	No	11 (Good)
Li X et al. (2023) (35)	Yes	Yes	Yes	Yes	No	Yes	Yes	Yes	Yes	Yes	Yes	NS	Yes	No	11 (Good)
Chunlan S et al. (2024) (36)	Yes	Yes	Yes	Yes	No	Yes	Yes	Yes	Yes	Yes	Yes	NS	Yes	No	11 (Good)
Delombaerde D et al. (2024) (37)	Yes	Yes	Yes	Yes	No	Yes	Yes	Yes	Yes	Yes	Yes	NS	Yes	No	11 (Good)

Q1: Was the research question or objective in this paper clearly stated?, Q2: Was the study population clearly specified and defined?, Q3: Was the participation rate of eligible persons at least 50%?, Q4: Were all the subjects selected or recruited from the same or similar populations (including the same time period)? Were inclusion and exclusion criteria for being in the study prespecified and applied uniformly to all participants?, Q5: Was a sample size justification, power description, or variance and effect estimates provided?, Q6: For the analyses in this paper, were the exposure(s) of interest measured prior to the outcome(s) being measured?, Q7: Was the timeframe sufficient so that one could reasonably expect to see an association between exposure and outcome if it existed?, Q8: For exposures that can vary in amount or level, did the study examine different levels of the exposure as related to the outcome (e.g., categories of exposure, or exposure measured as continuous variable)?, Q9: Were the exposure measures (independent variables) clearly defined, valid, reliable, and implemented consistently across all study participants?, Q10: Was the exposure(s) assessed more than once over time?, Q11: Were the outcome measures (dependent variables) clearly defined, valid, reliable, and implemented consistently across all study participants?, Q12: Were the out-come assessors blinded to the exposure status of participants?, Q13: Was loss to follow-up after baseline 20% or less?, Q14: Were key potential confounding variables measured and adjusted statistically for their impact on the relationship between exposure(s) and outcome(s)?, Good: Met 11–14 criteria, Fair: Met 6–10 criteria, Poor: Met 0–5 criteria. NIH, National Institutes of Health; NS, not specified.

## Discussion

### Main findings of the present systematic review

This systematic review, that was primarily designed to evaluate the effect of ICI therapy on biventricular mechanics in advanced cancer patients over a short-term follow-up period, revealed: 1) subtle changes (falling within normal ranges) of traditional indices of LV and RV systolic function and hemodynamics, assessed by conventional TTE; 2) a significant attenuation of all biventricular and biatrial myocardial strain parameters, compared to baseline values and to the accepted reference values; 3) a moderate increase in serum troponin levels, in absence of any significant changes in serum NT-proBNP levels; 4) a low-to-moderate prevalence of IRAEs, particularly pneumonitis, dermatitis, thyroiditis and myositis; 5) a moderate prevalence of CTRCD, affecting approximately one-third of ICI-treated patients; 6) a low prevalence of acute ICI-related myocarditis, with an estimated mortality rate of 42.8%.

Overall, ICI-treated cancer patients were mostly middle-aged males with a low-to-moderate cardiovascular disease burden, with no evidence of structural cardiomyopathy, with preserved biventricular systolic function and with normal hemodynamics at basal evaluation. They were more likely to be prescribed with PD-1 inhibitors, whereas PD-L1 inhibitors and CTLA-4 inhibitors were less commonly administered. Cardioprotective drugs, particularly beta blockers and ACEi/ARBs, were complessively underutilized in these patients. Serial assessment of conventional echoDoppler parameters excluded ICI-induced cardiotoxicity, whereas strain analysis highlighted a significant reduction of myocardial strain parameters magnitude over a short-term follow-up period. Similar findings were provided by STE and CMR-FT studies, indicating that myocardial deformation indices are more sensitive than conventional echocardiographic parameters for the early detection of ICI-related subclinical myocardial toxicity. No patient was lost to follow-up and myocardial deformation indices were adequately assessed by all the included studies, with excellent intra- and inter-rater reliability. Early impairment in biventricular mechanics was associated with moderate increase in serum levels of HS-troponin, as demonstrated by two-third of the included studies.

The four imaging studies that evaluated both LV and RV myocardial strain parameters (26, 30, 31, 34) demonstrated a concomitant attenuation of biventricular mechanics after ICI treatment, indicating that LV and RV systolic function functions were early injured simultaneously after ICIs administration. This finding was consistent with the assumption that left and right ventricular toxicity can occur simultaneously in patients undergoing oncology therapies (43). Both LV-GLS and RVFWLS/RV-GLS showed an incremental diagnostic value over conventional indices of biventricular systolic function, LVEF and TAPSE respectively. The pooled LV-GLS magnitude obtained by the strain imaging studies included in this systematic review was 16.8%, slightly reduced in comparison to the value of 20%, that is currently accepted as optimal cutoff value in healthy individuals (38, 44). The pooled relative decline in LV-GLS at follow-up time point compared to baseline was of 13.4%, whereas the pooled relative reduction in LVEF was only of 2.9%. With regards to RV systolic function, the reduction in RV-GLS and RV-FWLS values after ICI treatment were of 14.5% and 15.9% respectively, while TAPSE showed a decrease of only 6.9%.

Several factors may affect the accuracy and sensitivity of LVEF and TAPSE in detecting subclinical cardiac toxicity. LVEF assessment is strongly influenced by the echocardiographic imaging quality for optimal visualization of endocardial border (45), is based on geometric assumptions (15), is a load-dependent index (pre-load and after-load can affect the LVEF value) (15) and, most of all, is limited by the large inter-rater and test-retest variability (46). TAPSE, a surrogate marker of RV systolic function, is a mono-dimensional parameter which may not reflect the global RV systolic function; indeed, TAPSE is an angle-dependent and preload-dependent parameter, that may be increased in the presence of severe tricuspid regurgitation or preserved in the presence of mildly reduced RV systolic function (47). Differently from LVEF and TAPSE, LV-and RV myocardial strain parameters have relative angle independence and less load dependence advantages with good reproducibility (48).

### Pathophysiological mechanisms underpinning biventricular mechanics impairment in ICI-treated patients

As demonstrated by the CMR-FT studies included, ICI-treated patients may early develop diffuse myocardial edema, a possible correlate for an ICI-induced immune cell infiltration (4), and subsequently myocardial fibrosis, as a long-term consequence of ongoing myocardial inflammation (28). The pathophysiological changes affecting the extracellular matrix may impact myocardial mechanics by increasing myocardial stiffness (20–22). The greater is the myocardial stiffness, the lower is the magnitude of myocardial strain parameters assessed by imaging studies. This pathophysiological process may affect both ventricles and atria, as detected by the imaging studies included in this systematic review and as demonstrated in various clinical settings (49–52).

Two Authors (30, 31) observed that RV mechanics impairment occurred earlier than LV-GLS decline. This finding was attributed to the higher susceptibility of the right ventricle to the administration of ICIs, due to its smaller myocardial mass and thinner walls compared with the left ventricle (53). In addition, given that the interventricular septum is shared by the left ventricle and right ventricle and contributes to the mechanical function of both ventricles (54), the RV-GLS magnitude is related to both RV and LV systolic function.

Even if few data are available about the impact of ICIs on bi-atrial reservoir strain, it is likely that bi-atrial dysfunction may reflect disturbed biventricular mechanics. Myocardial edema and increased myocardial stiffness may cause reduction of bi-atrial reservoir strain in ICI-treated patients, as observed in conditions of chronic pressure overload (55, 56).

### Cellular and molecular basis of ICI-induced cardiotoxicity

During the last few years, some Authors have evaluated the pro-inflammatory and pro-fibrotic effects of short-term ICIs therapy in preclinical models. Quagliariello et al. (57) demonstrated that ICI treatment, using anti-PD1 and anti-CTLA4 agents, activated T-cell *in-vitro*, inducing a significant cardiomyocyte lysis that was associated with the release of damage-associated molecular pattern and pro-inflammatory cytokine, such IL-1α, IL-1β, IL-6, IL-17a and TNF-α. *In vivo*, using immunocompetent mice, the authors also demonstrated that short-term treatment with anti-PD-1 or anti-CTLA-4 drugs negatively affected radial and longitudinal strain, promoting cardiac fibrosis, through the up-regulation of galectin-3, pro-collagen 1-α and MMP-9. While both pembrolizumab and ipilimumab significantly increased vascular inflammation, a higher increase of NF-kB expression was observed in anti-PD1-treated animals compared with anti-CTLA-4 treated group. Accordingly, a significant hypertrophy, characterized by a considerable increase of the cytoplasmic volume together with an irregular course of the cardiomyocytes, was observed only in animals treated with pembrolizumab. Conversely, ipilimumab-treated mice showed a linear and consistent longitudinal morphology of cardiomyocytes, and no hypertrophy was found. In myocardial tissue, increased levels of G-CSF and GM-CSF, two growth factors contributing to immune cell differentiation and recruitment, and involved in heart failure and hypertrophy, and also other pro-inflammatory and pro-fibrotic cytokines, namely IL-6, IL17-α, IFN-γ, and TNF-α, were observed in ICI-treated group compared with untreated mice (57). Chen et al. (58) generated a mouse model of ICI-induced cardiotoxicity, using BMS-1, a specific PD-1/PD-L1 inhibitor. They found that the inhibition of PD1/PD-L1 axis in a mouse model of melanoma was associated to cardiomyocyte apoptosis and cardiotoxicity due to the alteration in gut microbiota. This dysbiosis was accompanied by low butyrate production, induction of macrophage polarization towards a M1-like phenotype and production of TNF-α and IL-1β via PPARα-CYP4X1 axis inhibition, all events associated with an increased release of myocardial enzymes (such CK-MB, AST, CK and LDH) (58). Moreover, Poels et al. (59) demonstrated that CTLA-4 promoted T-cell activation, decreasing naïve CD44^-^CD62L^+^CD4^+^ T-cells and, parallelly, increasing circulating CD44^+^CD62L^-^ CD4^+^ and CD8^+^ T-cells, thereby exacerbating atherosclerotic plaque inflammation and progression in Ldlr^-/-^ mice. Additionally, αCTLA4 treatment resulted in ICAM1 up-regulation, activating aortic endothelium, and promoting the formation of plaques characterized by a larger necrotic core and reduced collagen (59). Similar results were also observed using αPD-1/PD-L1 treatment that was found to heighten atherosclerosis process by fostering a T cell-driven inflammation (60). In a preclinical model of melanoma, anti-PD-1 immunotherapy was associated with the impairment of LV systolic function, leading to a decreased fractional shortening and ejection fraction. This phenomenon was correlated with the expansion of activated T-cells infiltrating the myocardium and with the significant increase of cleaved caspase-3 and mouse plasma cardiac troponin I expression levels in anti-PD-1 treated mice hearts compared with untreated animals (61). To better understand and study the cellular and molecular mechanisms that can contribute to ICI-related cardiotoxicity, in 2020 Wei and colleagues developed a robust model recapitulating ICI-induced myocarditis, characterized by the complete deletion of *Pdcd1* and the mono-allelic loss of *Ctla4.* In Ctla4^+/-^ Pdcd1^-/-^ mice, cardiomyocyte necrosis, and severe electrocardiographic abnormalities were associated with the increased infiltration within the myocardium by T cells and macrophages, highlighting the role of the iper-activation of the immune system in the pathogenesis of ICI-related myocarditis (62).

### Acute ICI-related myocarditis

The estimated pooled prevalence of acute ICI-associated myocarditis (0.8%) was in alignment with literature data (8, 9, 63). This serious complication more commonly occurs about 1 month after receiving ICI therapy (60) and may be fulminant in 25-50% of patients (10–12). The combination of anti-PD-1 and anti-CTLA-4 antibodies appears to be associated with a potentially higher risk of myocarditis, as well as an increased likelihood of fatal outcomes compared to ICI monotherapy, while it remains unclear whether the risk is similarly elevated with the combination of chemotherapy and immunotherapy, given the limited data available (64–66). Males are affected by ICI-related myocarditis in approximately two-third of cases (67). Moreover, myocarditis is more common in patients with melanoma (67). The pathophysiological mechanism underpinning ICI-induced myocarditis is not fully understood. Two principal mechanisms may be involved in this process. One is the breakdown of immune tolerance to the heart mediated by the CTLA-4, PD-1, and LAG-3 pathways, and the other involves the expansion of T cells targeting a common antigen shared by the cancer and the heart (68, 69). Histologically, ICI-associated myocarditis is associated with infiltration of CD4^+^ and CD8^+^ T cells and CD68^+^ macrophages into the myocardium and conduction system (68, 70).

Clinically, ICI-associated myocarditis is arrhythmogenic and is associated with myositis and a myasthenia-gravis-like syndrome, likely due to T cell targeting of a shared antigen between skeletal and cardiac muscle (71, 72). In addition, ICI-treated cancer patients seem to have a 3-fold higher risk for cardiovascular events (myocardial infarction, coronary revascularization, and ischemic stroke) (73). The diagnosis of myocarditis can be aided by elevated serum levels of myocardial markers, such troponin and NTpro-BNP. TTE is an important imaging tool for patients with suspected ICI-related myocarditis; however, LVEF can be normal in at least half of patients with ICI-associated myocarditis (8, 74). On the other hand, LV-GLS assessed by strain echocardiographic imaging is reduced among patients with ICI-associated myocarditis presenting with both a preserved and reduced EF and a lower GLS magnitude is strongly associated with major adverse cardiac events in ICI myocarditis (75). CMR is the preferred imaging modality for diagnosing myocarditis, allowing to identify the characteristics of fibrosis and inflammatory tissues in the early stages of the disease; abnormal values in T1 and T2 mapping of CMR may provide significant diagnostic value (76). Myocardial biopsy is the gold standard for diagnosis; pathological examination usually reveals T-lymphocyte and macrophage infiltration as well as the death of cardiomyocytes (77). Drug discontinuation and high dose corticosteroid therapy are the most important treatments of ICI-related myocarditis (24, 78). If corticosteroid therapy is ineffective, other immunosuppressants, such as mycophenolate mofetil, anticalcineurin, anti-thymocyte globulin, or intravenous immunoglobulin, may be administered (5, 8).

It is noteworthy that ICI therapy may also induce other cardiovascular IRAEs, including vasculitis (79), pericarditis (80), and arrhythmias, such as supraventricular arrhythmias, ventricular arrhythmias, and conduction disturbances (81).Also in this case, the risk of serious cardiovascular IRAEs is higher with the dual checkpoint inhibition (anti-PD-1/PD-L1 and anti-CTLA-4) than with monotherapy (9).

### Implications for clinical practice

The results of this systematic review confirm the usefulness of strain imaging for detecting subclinical cardiotoxicity in cancer patients. LV-GLS assessment is a validated and highly reproducible indicator of LV systolic function (82). It represents a sensitive measure of cardiac function and cardiac injury (83, 84), given that deformation parameters can detect early systolic impairment in the presence of preserved LVEF (≥55%) (85, 86). GLS measurement improves the prognostic risk stratification and may help the clinicians to select the most appropriate treatment in asymptomatic LV dysfunction caused by several etiologies (87–89). Determination of LV-GLS is recommended by the 2022 ESC Guidelines at baseline, particularly in moderate- and high-risk patients receiving anthracyclines and/or trastuzumab (24). A median GLS change of 15% is the threshold recommended when monitoring GLS during cancer therapy (24). Considering that the cardiotoxicity of immunotherapy can involve both LV and RV systolic function, a comprehensive assessment of biventricular mechanics by strain imaging should be implemented in the clinical practice. Strain imaging should be performed before starting ICI therapy and possibly every two weeks during the first two months of treatment. Early detection of LV and/or RV-GLS impairment in ICI-treated patients, despite preserved biventricular systolic function on conventional TTE, may prompt clinicians to consider early intervention, such as early corticosteroid treatment, up-titrating cardioprotective medications and/or ICI discontinuation, to prevent myocarditis and other serious cardiovascular IRAEs. However, this should be discussed on a case-by-case basis, as no definitive guidelines are available to recommend an optimal management strategy.

### Limitations of the included studies

Main limitations of the included studies were the monocentric nature for 83.3% of total, the retrospective design for 41.7% of total and the lack of adjusted data for 66.7% of total. Moreover, the included studies were significantly heterogeneous in relation to the cancer type, the specific ICI therapy administered, the specific software used for measuring myocardial strain parameters and finally the follow-up duration. With regards to the strain imaging method employed by the included studies, a number of strengths and limitations of CMR-FT and strain echocardiographic imaging should be acknowledged. CMR-FT provides the most accurate and reproducible assessments of global ventricular volumes and cardiac function, but is limited by suboptimal temporal resolution, high associated costs, low availability and the time-consuming nature (90). Strain echocardiographic imaging has higher temporal resolution and is more widely available than CMR-FT, but strain measurements may be subject to inter-vendor variability and are strongly dependent on good image quality, on frame rate (low frame rates are associated with the loss of speckles and accuracy), on loading conditions and finally on extrinsic mechanical factors, such as the chest wall conformation (91–94).

## Conclusions

The ICI treatment causes a significant deterioration of biventricular mechanics, early diagnosed by strain imaging methods. Myocardial strain parameters are more sensitive than conventional indices of systolic function for the early detection of subclinical cardiotoxicity.

Comprehensive assessment of cardiac function by strain imaging analysis should be implemented in the clinical practice for monitoring ICI-related cardiotoxicity. Further studies should be designed to evaluate if early introduction and/or up titration of cardioprotective therapy might prevent and/or attenuate ICI-associated cardiovascular IRAEs complications.

## Data Availability

The original contributions presented in the study are included in the article/supplementary material. Further inquiries can be directed to the corresponding authors.
